# Pregnancy After Laparoscopic Radiofrequency Ablation: A Case Report

**DOI:** 10.1097/og9.0000000000000209

**Published:** 2026-10-13

**Authors:** Alberto Sirven, Liz Yanes

**Affiliations:** West Kendall OBGYN, Miami, Florida; and Iberoamerican University, Miami, Florida.

## Abstract

**BACKGROUND:**

Laparoscopic radiofrequency ablation (RFA) has garnered popularity as a promising uterus-sparing option for the management of leiomyomas. However, data on fertility and reproductive outcomes, particularly rare complications such as uterine rupture, remain limited.

**CASE:**

Twenty-nine-year-old G0 patient with infertility underwent uncomplicated laparoscopic RFA of a 1.18×1.33–cm anterior fundal intramural leiomyoma. She conceived spontaneously 2 years later and presented at 17 weeks of gestation with an anterior fundal uterine rupture, which was repaired successfully. Patient underwent scheduled cesarean delivery at 35 weeks and delivered a live-born 2,390-g boy with Apgar scores of 9 at 1 minute and 9 at 5 minutes. Pelvic magnetic resonance imaging at 6 months postpartum (26 months after ablation) revealed no myometrial lesions.

**CONCLUSION:**

Current data on reproductive outcomes after laparoscopic RFA are reassuring with regard to uterine rupture, but more prospective studies are needed. Although rare, uterine rupture should be considered a potential serious risk after laparoscopic RFA and discussed during patient counseling.

## Abstract 2

Laparoscopic radiofrequency ablation is a promising uterus-sparing leiomyoma treatment, but pregnancy outcome data are limited.

**Teaching Points**

1. Only 69 cases of pregnancy after laparoscopic radiofrequency ablation (RFA) management of leiomyomas exist in the literature. The existing data are limited in scope and potentially underestimate the true incidence of complications and adverse outcomes.
2. Clinicians should recognize the possibility of uterine rupture as a rare but potentially fatal complication that might arise after laparoscopic RFA, warranting its inclusion in comprehensive patient counseling.

Leiomyomas are the most common gynecologic neoplasm, with most sources estimating that up to 70% of women will have at least one by menopause.^1^ Leiomyomas are a frequent cause of abnormal uterine bleeding (AUB), bulking symptoms (pelvic pain, constipation, urinary dysfunction), infertility, anemia, and low back pain.^2–4^

Although hysterectomy is the only definitive treatment for leiomyomas, many women seek to conserve the uterus.^5^ The reason may be incomplete fertility, fear of surgery, or personal beliefs.^6^ This can be achieved through laparoscopy, hysteroscopy, uterine artery embolization, magnetic resonance–guided high-intensity ultrasound, high-intensity focused ultrasound, and RFA.^7–9^

Among these, RFA decreases uterine leiomyoma burden by inducing coagulative necrosis in a precise manner using ultrasound guidance.^2^ Radiofrequency ablation can be performed transvaginally with the Sonata system or laparoscopically with the Acessa procedure.^10^ Laparoscopic RFA has lower theoretical risk of endometrial damage, which makes it particularly attractive for women desiring future fertility.^10,11^

Laparoscopic RFA has demonstrated a lower reintervention rate (0.3%) and the lowest major complication rate (1.7%) compared with other uterine-sparing surgical interventions, although comparative data are limited.^8^ There is, however, a paucity of data on fertility and reproductive outcomes, especially regarding rare but serious complications such as uterine rupture, which may underestimate the true incidence of complications and undesirable outcomes.^5,8,12,13^ We report a case of uterine rupture after laparoscopic RFA, a rare but life-threatening complication, and synthesize the limited available evidence.

A narrative review of the literature was performed and completed on May 2026 by searching PubMed and Google Scholar for the key words “radiofrequency ablation fibroids pregnancy,” “Acessa pregnancy outcome,” “uterine rupture radiofrequency ablation fibroids,” and “pregnancy outcome radiofrequency ablation.” We identified 68 pregnancies after laparoscopic RFA for leiomyomas (Table 1). Only peer-reviewed articles published in English were included; duplicates were removed.

**Table 1.** Pregnancy Outcomes After Laparoscopic Radiofrequency Ablation Management of Uterine Leiomyomas

Study	Pregnancies (n)	Live Births (n)	1st-Term Miscarriages (n)	Method of Delivery	Complications
Berman et al^14^	6	5	1	1 cesarean delivery 4 vaginal deliveries	None reported
Polin and Hur^15^	40	32	5	16 cesarean deliveries 16 vaginal deliveries	1 placenta previa 1 postpartum hemorrhage
Allen et al^16^	22	11	10	6 cesarean deliveries 5 vaginal deliveries	1 intrauterine fetal death at 30 wk of gestation 1 uterine rupture during misoprostol treatment for miscarriage at 10 wk

## CASE

A 29-year-old G0 patient presented to our clinic in November 2023 for AUB and inability to conceive. The patient’s medical history and surgical history were noncontributory. A workup for AUB revealed a fundal intramural leiomyoma (leiomyoma 1) and a subserosal pedunculated leiomyoma (leiomyoma 2), both in the anterior wall of the uterus, measuring 1.18×1.33 cm and 2.16×1.7 cm, respectively; a vascular echogenic structure within the endometrium resembling a polyp was also noted.

On discussion of possible causes of AUB, the absence of alternative identifiable causes, and management options, the patient selected surgical uterine-sparing treatment. Myomectomy was considered for leiomyoma 1, but its intramural location meant myometrial incision, and closure would carry a recognized risk of scar dehiscence or rupture in future pregnancy; laparoscopic RFA was therefore selected to avoid a formal hysterotomy.

In December 2023, laparoscopic RFA was performed on leiomyoma 1 after its location (anterior superior fundus) and dimensions (2 cm transverse, 2 cm anterior–posterior, and 2 cm sagittal) were mapped through laparoscopic ultrasound. With ultrasound guidance, the tip of the handpiece was inserted with minimal pressure using rotational back-and-forth movement, penetrating the leiomyoma in a controlled fashion. The entry to the leiomyoma was limited to 1–2 cm, and its correct position was confirmed by ultrasound from all three planes. The retractable array of seven needle electrodes was then deployed from the handpiece tip into the surrounding leiomyoma tissue, delivering two treatments of 1.5 minutes of radiofrequency after tip ramp-up. Temperatures at the 7 needle electrodes were monitored continuously and averaged 100°C. The return pad temperature remained below 40°C. The electrodes were retracted; the system was switched to 8-W coagulation; and the probe was withdrawn. Laparoscopic excision was performed for leiomyoma 2 with monopolar electrosurgery on its base. Of note, the ablation treatment volume (2 cm in all planes) exceeded the dimensions of the leiomyoma itself (1.18×1.33 cm), consistent with standard RFA technique. Endometrium and ovarian implants were sampled. Procedure time was 1.5 hours; hemostasis was confirmed; and no complications were noted. Final pathology revealed benign proliferative endometrium (no discrete polyp), hemosiderin-laden macrophages within the ovary, endometriosis of the cul-de-sac, and leiomyoma, consistent with leiomyoma 2.

In December 2025 the patient presented to our office for pregnancy confirmation after conceiving spontaneously at 5 5/7 weeks of gestation. At this time, pelvic ultrasound revealed no leiomyomas.

At 17 5/7 weeks of gestation, the patient presented to the emergency room with acute abdomen and hypotension. On arrival, the patient underwent fluid resuscitation, fetal monitoring, and intravenous access placement. An obstetric ultrasound was limited but revealed a healthy fetus. Right upper quadrant ultrasound revealed hemoperitoneum with septations, prompting a pelvic magnetic resonance imaging (MRI), which showed possible uterine rupture. The patient underwent emergent laparotomy, confirming a 4- to 5-cm defect in the mid anterior fundal wall. Hemoperitoneum 600 cm^3^ was evacuated; amnioreduction was performed (120 cm^3^) to reduce the bulging amniotic bag, followed by myometrium closure with one running layer of 0 vicryl, followed by a second layer of 0 vicryl to imbricate the first layer. Anatomy scan at 19 weeks of gestation revealed 3.5-cm myometrial thickness over the area of hysterorrhaphy.

The patient was discharged on postoperative day 4 with instructions for strict bed rest, followed by biweekly outpatient ultrasounds and visits to monitor uterine wall thickness and fetal well-being. The remainder of the pregnancy was uneventful aside from diet-controlled gestational diabetes and a short cervix (2.0 cm) managed with vaginal progesterone. Given the paucity of data on recurrent uterine rupture risk and decision–analytic modeling proposing delivery between 34 and 36 weeks in women with prior uterine rupture, a multidisciplinary team scheduled cesarean delivery at 35 weeks.^17^ The patient received steroids at 34 weeks and delivered a live-born 2,390-g boy, with Apgar scores of 9 at 1 and 9 at 5 minutes, at 35 weeks by scheduled cesarean. There were no postpartum complications.

Pelvic MRI at 6 months postpartum (26 months after ablation) revealed no myometrial lesions and no persistent myometrial defect, with susceptibility artifact noted at the anterior fundus in keeping with prior intervention.

## DISCUSSION

The precise mechanism responsible for rupture in this case cannot be established with certainty, although several hypotheses can be considered. Although laparoscopic RFA is generally believed to preserve uterine integrity, it is conceivable that alterations in targeted myometrial architecture could predispose susceptible patients to rupture during pregnancy. Of note, the ablation volume in this case exceeded the dimensions of the leiomyoma and thus necessarily included a margin of adjacent normal myometrium, offering a more concrete anatomic basis for this hypothesis. That the rupture occurred in the anterior fundal region, the location of the treated leiomyoma, suggests a possible relationship between prior ablation and subsequent uterine wall failure. The 2-year interval between intervention and rupture suggests that any alterations on myometrial integrity may persist beyond the immediate postoperative period. However, several limitations should be acknowledged. Histopathologic evaluation of the rupture site was not available, precluding direct assessment of tissue changes attributable to prior ablation. Imaging obtained before laparoscopic RFA documented uterine and leiomyoma dimensions but did not include a measurement of myometrial thickness at the site of leiomyoma 1, so no baseline value is available for comparison. Similarly, the pregnancy confirmation ultrasound did not document gestational sac implantation relative to the ablation site, limiting assessment of whether implantation proximity to the treated myometrium contributed to the rupture. Postpartum pelvic MRI demonstrated no myometrial lesions or persistent myometrial defect but did not report a specific myometrial thickness measurement at the site of repair, limiting assessment of long-term structural integrity at this site. Furthermore, causality cannot be established from a single case.

The original clinical trials excluded women who desired future fertility, so no pregnancy outcome data existed at the time of approval by the U.S. Food and Drug Administration.^12,13^ However, six subjects unexpectedly conceived; five had full-term deliveries, and one had a first-trimester miscarriage.^14^

A retrospective review of 40 pregnancies after laparoscopic RFA procedure showed no cases of uterine rupture, invasive placenta, abruption, or fetal growth restriction, although one placenta previa and one postpartum hemorrhage occurred.^8,15^ A prospective cohort study (ULTRA [Uterine Leiomyoma Treatment With Radiofrequency Ablation] Study) described 22 pregnancies after laparoscopic RFA, 10 of which ended in first-trimester miscarriage, with one intrauterine fetal demise, and 11 live births.^16^ Of note, one of the miscarriages was complicated by a uterine rupture during misoprostol treatment at 10 weeks.^16^ In our review, we could not find any reports of uterine rupture in an ongoing pregnancy after laparoscopic RFA.

Although the existing data on uterine rupture after laparoscopic RFA are reassuring, this should not be extended to reproductive outcomes overall. Aggregated data from the reviewed studies show a miscarriage rate of approximately 24% (16 of 68), which is not negligible, and highlight that the absence of reported rupture should not be conflated with an absence of reproductive risk more broadly. Cases like this challenge the notion that laparoscopic RFA is uniformly benign for future pregnancies, although limited by its nature as a single report. Clinicians should nonetheless recognize uterine rupture as a rare but potentially fatal complication after laparoscopic RFA, warranting inclusion in patient counseling and underscoring the need for further research.

## References

[1] Grube M Neis F Brucker SY Kommoss S Andress J Weiss M . Uterine fibroids–current trends and strategies. Surg Technol Int 2019;34:257–63. 30888674

[2] Management of symptomatic uterine leiomyomas. ACOG Practice Bulletin, Number 228. American College of Obstetricians and Gynecologists. Obstet Gynecol 2021;137:e100–15. doi: 10.1097/AOG.0000000000004401 34011888

[3] George JW. The burden of uterine fibroids: an overview. J Histotechnol 2023;46:153–5. doi: 10.1080/01478885.2023.2265185 37791571

[4] Fortin C Flyckt R Falcone T. Alternatives to hysterectomy: the burden of fibroids and the quality of life. Best Pract Res Clin Obstet Gynaecol 2018;46:31–42. doi: 10.1016/j.bpobgyn.2017.10.001 29157931

[5] Vannuccini S Petraglia F Carmona F Calaf J Chapron C. The modern management of uterine fibroids-related abnormal uterine bleeding. Fertil Steril 2024;122:20–30. doi: 10.1016/j.fertnstert.2024.04.041 38723935

[6] Ghezzi F Cromi A Bergamini V Scarperi S Bolis P Franchi M. Midterm outcome of radiofrequency thermal ablation for symptomatic uterine myomas. Surg Endosc 2007;21:2081–5. doi: 10.1007/s00464-007-9307-8 17514400

[7] Berman JM Shashoua A Olson C Brucker S Thiel JA Bhagavath B. Case series of reproductive outcomes after laparoscopic radiofrequency ablation of symptomatic myomas. J Minim Invasive Gynecol 2020;27:639–45. doi: 10.1016/j.jmig.2019.06.009 31238151

[8] Lewis JM Algurjia E Lumsden MA Meinhold-Heerlein I Wasson MN Spiers A . Image-guided therapies for uterine fibroids. Int J Gynaecol Obstet 2025;171:495–506. doi: 10.1002/ijgo.70493 40888469

[9] Iversen H Dueholm M. Radiofrequency thermal ablation for uterine myomas: long-term clinical outcomes and reinterventions. J Minim Invasive Gynecol 2017;24:1020–8. doi: 10.1016/j.jmig.2017.05.021 28662989

[10] Lee BB Yu SP. Radiofrequency ablation of uterine fibroids: a review. Curr Obstet Gynecol Rep 2016;5:318–24. doi: 10.1007/s13669-016-0183-x PMC5114324 27917310

[11] Wong WSF. New surgical approach to treat fibroids and solid tumors–thermal and nonthermal ablation. Gynecol Minim Invasive Ther 2023;12:191–4. doi: 10.4103/gmit.gmit_18_23 PMC10683962 38034109

[12] Galen DI Pemueller RR Leal JG Abbott KR Falls JL Macer J. Laparoscopic radiofrequency fibroid ablation: phase II and phase III results. JSLS 2014;18:182–90. doi: 10.4293/108680813X13693422518353 PMC4035627 24960480

[13] Keltz J Levie M Chudnoff S. Pregnancy outcomes after direct uterine myoma thermal ablation: review of the literature. J Minim Invasive Gynecol 2017;24:538–45. doi: 10.1016/j.jmig.2017.01.009 28109894

[14] Berman JM Bolnick JM Pemueller RR Garza Leal JG. Reproductive outcomes in women following radiofrequency volumetric thermal ablation of symptomatic fibroids: a retrospective case series analysis. J Reprod Med 2015;60:194–8. 26126303

[15] Polin M Hur HC. Radiofrequency ablation of uterine myomas and pregnancy outcomes: an updated review of the literature. J Minim Invasive Gynecol 2022;29:709–15. doi: 10.1016/j.jmig.2022.01.015 35123041

[16] Allen A Schembri M Parvataneni R Waetjen LE Varon S Salamat-Saberi N . Pregnancy outcomes after laparoscopic radiofrequency ablation of uterine leiomyomas compared with myomectomy. Obstet Gynecol 2024;143:612–8. doi: 10.1097/AOG.0000000000005548 38422502

[17] Frank ZC Lee VR Hersh AR Pilliod RA Caughey AB. Timing of delivery in women with prior uterine rupture: a decision analysis. J Matern Fetal Neonatal Med 2021;34:238–44. doi: 10.1080/14767058.2019.1602825 30935266

